# 1805. Safety Outcomes Following Beta-Lactam use in Patients with a Documented Beta-Lactam Allergy

**DOI:** 10.1093/ofid/ofac492.1435

**Published:** 2022-12-15

**Authors:** Daniel Rampersad, Tyler Maxwell, Stanley Moy

**Affiliations:** One Brooklyn Health - Brookdale University Hospital Medical Center, Brooklyn, New York; Touro College of Pharmacy, New York, New York; SUNY Downstate Health Sciences University, Brooklyn, New York

## Abstract

**Background:**

Management of bacterial infections typically consist of beta-lactam antibiotics. Individuals with histories of beta-lactam allergy are more likely to receive alternative broad-spectrum antibiotics which may be less efficacious or lead to increased adverse events. Patients can be prescribed full doses of structurally non-related beta-lactam antibiotics due to the low similarity of side chains between these medications and penicillin, and the low incidence of penicillin allergies being true allergies. Nevertheless, there is a lack of data regarding safety outcomes in patients who are treated with a beta lactam with a documented allergy.

**Methods:**

This was a single-center retrospective observational study of patients with a documented beta-lactam allergy who received at least one dose of any beta-lactam antimicrobial excluding aztreonam from SUNY Downstate Health Sciences University between January 2015 – October 2021. Electronic medical records were reviewed for relevant study data. The primary outcome was the incidence of allergic reaction on full dose beta lactam with a documented allergy in the medical record.

**Results:**

Baseline characteristics are summarized in Table 1.

Two patients experienced a suspected allergic reaction while on beta-lactam therapy. Anaphylaxis was not reported and pharmacologic rescue agents were not required for each case. The antimicrobial agent was changed after the reaction was noted in both cases.

**Figure:**
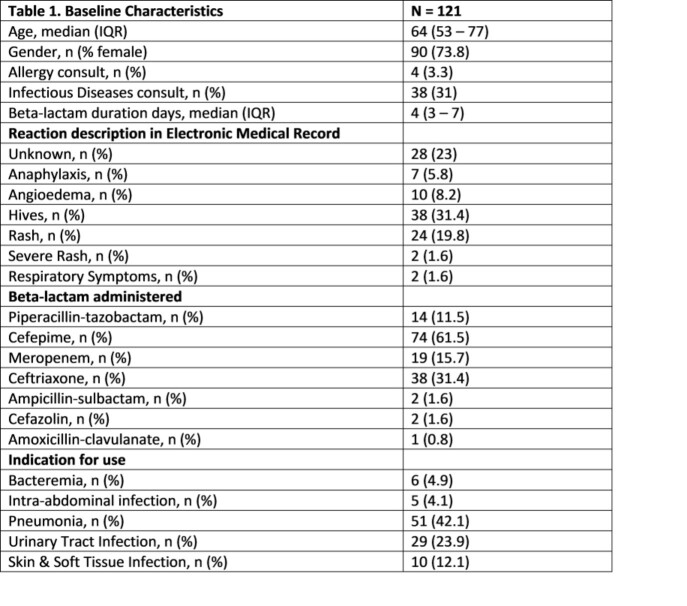

**Figure:**
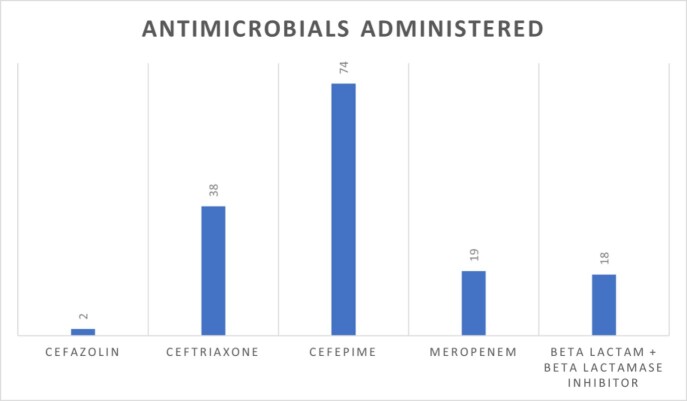

**Figure:**
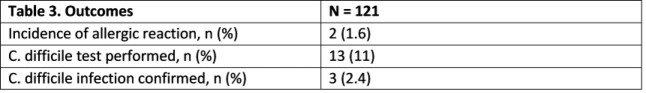

**Conclusion:**

Patients who were administered beta-lactam antimicrobials with beta-lactam allergy had low rates of allergic reaction. The rate of allergic reactions observed is similar to the incidence of allergy in patients without documented allergies reported in the literature. Administration of beta-lactams with dissimilar side chains such as beta-lactam beta-lactamase inhibitors and third and fourth generation cephalosporins were safely administered to patients with a documented allergy.

**Disclosures:**

**All Authors**: No reported disclosures.

